# Evaluation of polyherbal formulation in broilers fed high energy diet: Implications on zootechnical parameters, fat accretion, and serum L-carnitine levels

**DOI:** 10.5455/javar.2022.i581

**Published:** 2022-03-13

**Authors:** Prashanth D’souza, Ramasamy Selvam

**Affiliations:** 1 Formulation and Development, R & D Centre, Natural Remedies Private Limited, Bangalore, India; 2 Technical Service, R & D Centre, Natural Remedies Private Limited, Bangalore, India

**Keywords:** Chickens, choline chloride, fat accretion, high energy diet, polyherbal formulation, serum L-carnitine

## Abstract

**Objective:**

The current broiler trial was planned to assess the effects of Kolin Plus™, a polyherbal formulation (PHF), on performance, protein and fat accretion, and serum L-carnitine (LC) levels in broilers fed a high-energy diet (HED).

**Materials and Methods:**

A total of 500 1-day-old Cobb 430 male chicks were assigned to 5 treatment groups consisting of 10 replicates, with 10 birds in each replicate (*n* = 100). Group G1 was a negative control fed HED, and group G2, a positive control supplemented with synthetic choline chloride (SCC) 1,500 gm/ton in HED. Groups G3, G4, and G5 were supplemented with PHF in HED at 400, 500, and 750 gm/ton feed, respectively (PHF400, PHF500, and PHF750).

**Results:**

The PHF produced a dose-dependent numerical improvement in body weight, feed conversion ratio, livability, and the European Production Index. There were no changes in carcass nitrogen and protein accretion, whereas a statistically significant decrease (*p* < 0.05) in carcass fat and fat accretion was observed in the SCC and PHF groups. Moreover, PHF showed a significant increase in serum LC levels.

**Conclusion:**

Kolin Plus™ improves performance parameters akin to SCC by improving fat metabolism and mobilization by enhancing serum LC levels and restoring normal fat accretion.

## Introduction

High-energy diets (HED) have been utilized to shorten the rearing period and increase profitability in poultry, especially during the finisher period, and are known to predispose broiler chickens to fatty liver syndrome (FLS) [1], higher abdominal fat [2], footpad problems [3], and pulmonary hypertension [4]. These metabolic syndromes generally affect fast-growing broilers fed HED due to the methyl group donors’ deficiency in feed, culminating in excessive lipid accretion in the hepatic cells followed by liver damage [5]. This statement supported the findings that a high-calorie and low-protein diet is a prerequisite to initiating metabolic disorders in chickens [6]. The accumulation of extra fat in the abdominal and visceral areas of meat is a big problem for meat producers because fat is a waste that most people do not like. Meat producers have taken many steps to keep fat from building up, but they have had mixed results [7].

Supplementation of lipotropic agents along with HED has been practiced to alleviate the detrimental effects of HED on liver health and prevent metabolic disorders. Choline is an essential lipotropic agent that prevents the accumulation of excess fat and fatty liver in chickens [8] and plays a significant role in the synthesis of methionine from homocysteine as a labile methyl donor [9]. Additionally, it is crucial for the structural maintenance of cell membranes and organelles, proper maturation of the cartilage matrix of the bones [10], and a precursor molecule for the formation of acetylcholine in the nervous system [8]. Previous studies have endorsed the addition of choline chloride in poultry diets for augmented performance parameters such as feed conversion ratio (FCR) during the finisher period and alleviated fat deposition in the abdomen of broiler chickens [11]. In addition, it was said that broiler chickens are given choline and/or L-carnitine (LC) had less FLS [12].

Choline chloride is a prevalent form of synthetic choline that is added to animal feed and possesses certain limitations to it, such as being hygroscopic by absorbing atmospheric moisture, causing the generation of trimethylamine in the gut, and accelerating the loss of essential vitamins in the feed through oxidation [13]. Thus, researchers around the globe are focusing their efforts on the addition of herbal additives to broiler diets to counter the drawbacks of synthetic choline chloride (SCC). Several herbs and their phytochemical constituents have been employed as a substitute for SCC in animal diets and proven to exhibit activities akin to synthetic choline in poultry [8,. 

Recently, LC gained popularity due to its critical role in fatty acid degradation, as the transfer of long-chain fatty acid into the mitochondria is dependent on carnitine enzymes in food-producing animals. Diets enriched with L-carnitine reduce the fat accumulation in adipose tissue by enhancing fatty acid oxidation [15]. In addition, it has already been demonstrated that higher serum LC levels could be correlated with better meat quality in pigs [16].

Kolin Plus^TM^ is a polyherbal formulation (PHF) made by Natural Remedies Private Limited, a company in Bengaluru, India. It is a combination of parts of two plants, Acacia nilotica and Curcuma longa. Extracts of these individual herbs are hepatoprotective, antioxidant, and lipotropic *in vitro* and live systems [17–20]. The efficacy of Kolin Plus^TM ^was demonstrated in the choline deficiency model earlier [8], yet no scientific reports representing their efficacy in combination with poultry-fed HED are available. Hence, the current study aims to comparatively evaluate the effects of PHF and SCC on growth performance, protein and fat accretion in the carcass, and serum LC levels of Cobb 430 broiler chickens fed HED.

## Materials and Methods

### Institutional animal ethics committee approval

The study was carried out in concordance with the Institutional Animal Ethics Committee (IAEC Number: NR/AHS/PLT/12) guidelines. 

### Experimental design and feeding levels

A male flock of 500 Cobb 430 broiler chickens was randomly allotted to five groups. Group G1 was a negative control (NC) fed HED, and group G2, a positive control, supplemented with 1,500 gm/ton SCC in HED. Groups G3, G4 and G5 were supplemented with PHF in HED at 400, 500, and 750 gm/ton feed doses, respectively (PHF400, PHF500, and PHF750) to determine the optimum dose of PHF. Each dietary group had 10 replicate pens, and each pen housed 10 birds. The trial was conducted for 42 days. The birds were fed with a starter feed (1–14 days), a grower feed (15–28 days), and a finisher feed (29–42 days) in mash form, with an ad libitum supply of drinking water. The experimental NC diet (HED) was formulated by keeping the specifications close to the prevailing industry standard, with slightly higher metabolizable energy levels and moderately lower choline content levels in the starter, grower, and finisher phases. The experimental feed’s ingredient composition and nutritional values are presented in Tables 1–3.

### Experimental setup

A polyvinyl chloride brooder (PVC), a bell-style water drinker, a feeder for chicks, and/or a jumbo feeder for the chicken were provided in all pens. The floor space and size of the experimental pen were modified based on the total number of chicks allotted in each pen, using a chick-guard made of PVC. The shed temperature was regulated between 32°C and 34°C for the first 7 days, and then gradually abridged by 2°C every 7 days until 42 days. The relative humidity of the poultry house was maintained at 40%–70% during the study period. Standard vaccination and management practices were followed throughout the experiment for all the dietary groups. The vaccinations included those against the infectious bronchitis disease, administered at 0 day of age (IB Ma5) at the hatchery itself, Newcastle disease (live vaccine, Clone 30) at 5 and 20 days of age, and infectious bursal disease at 12 days of age (228E; Int plus). The lighting program included 24 h of light during the first week of age and 20 h of light after that, till the completion of the trial.

### Evaluation of zootechnical parameters

On days 1, 35, and 42, pen-wise body weight (BW) of the birds was recorded at the same time of the day without any fasting. A measured quantity of feed was offered to each pen every day in two equal divisions. Cumulative feed intake (CFI) was determined by deducting the number of orts left in each pen from the quantity of total feed offered. Then the average daily feed intake (ADFI) was determined. FCR was calculated as a ratio between ADFI and average body weight gain (BWG). Mortality was recorded as and when it happened, and the weight of the dead birds was recorded to adjust the data accordingly. European Production Index (EPI) was calculated according to the following formula:

EPI = [(100 – mortality) × (mean live weight/age) × 100]/FCR.

### Measurement of protein and fat accretion in the carcass

At the beginning of the experiment (day-1), 10 chicks were randomly selected and euthanized. The entire breast muscle was separated, minced, and triturated, following which it was subjected to an analysis of nitrogen (N2) and crude fat. The values obtained in this way were considered as base-level values. At 42 days, five birds were randomly selected from each group and killed by mechanical stunning, which was followed by cervical dislocation. The carcass was allowed to bleed out. It was then scalded at 55°C with intermittent dipping for 20 sec at a time, and the feathers were plucked up using de-feathering equipment. Next, the carcasses were manually eviscerated and rinsed. The breast muscle was separated; half of the entire breast was minced, triturated, and subjected to analysis for N2 and crude fat. Protein (N2 × 6.25) and fat accretion were calculated based on the difference in the base level values that were used as the common source of origin for all the dietary treatments.

**Table 1.** Feed formula—starter (HED—NC group).

Feed ingredients	Quantity (gm/kg)	Calculated nutrient value	
Maize	615.00	ME kcal/kg	3,000.0
Soybean meal 50	320.00	Crude protein %	22.0
De-oiled rice bran	10.70	Digestible amino acids %	
Rice bran oil—Starter^a^	16.00	Lysine	1.25
Di-calcium-phosphate	15.00	Methionine	0.60
Limestone powder	7.50	Methionine + Cysteine	0.94
DL-Methionine	3.00	Threonine	0.82
L-lysine HCl	3.20	Tryptophan	0.24
L-threonine	1.50	Arginine	1.30
Salt	2.40	Isoleucine	0.80
Sodium-bi-carbonate	2.00	Valine	0.90
Trace mineral premix^b^	0.50	Calcium %	0.80
Antibiotic Growth promoter	0.50	Available P %	0.48
Vitamin Premix^c^	0.50	Crude fiber %	2.92
Toxin Binder	1.00	Crude fat %	4.90
Mould inhibitor	0.50	Sodium %	0.22
Salinomycin 12%	0.50	Chloride %	0.20
Phytase 5000 EC	0.10	Potassium %	0.93
NSPase	0.10	Choline ppm	1,350

SCC and PHF at defined doses were added by replacing the equal quantity of de-oiled rice bran in HED.

a ME value assigned to rice bran oil was 8400 kcal/kg.

The ^b^mineral and ^c^vitamin premix were prepared as per the composition [8].

### Determination of serum L-carnitine levels

The serum was separated by spinning the clotted blood at 3,000 rpm for 5 min. Following the L-carnitine assay kit (Bioassay Technology Laboratory, Shanghai, China), 40 µl of samples and 10 µl of anti-L-carnitine antibodies were added to the sample wells. This was followed by adding 50 µl of streptavidin-HRP to the sample and standard wells; the solution was mixed thoroughly and incubated at 37°C for 1 h. Then the microplate was washed five times by soaking it in wells with wash buffer for 30 sec between each wash and blotted onto absorbent paper. Substrate solutions A and B were added in quantities of 50 µl each to all wells, and the plate was incubated for 10 min at 37°C in the dark. The reaction was then terminated using a stop solution (50 µl) and immediately measuring the optical density (OD) at 450 nm.

### Statistical analysis

For data related to performance traits, the replicates were considered the experimental units, while for the remaining data, individual observations were considered a single experimental unit. The raw data were cleaned up and presented as the mean of 10 replicates in each group. They then went through a one-way analysis of variance and the Tukey’s B Test (IBM SPSS Statistics Version 21.0; SPSS Inc., Chicago, IL).

## RESULTS

### Impact of PHF on zootechnical parameters in broilers fed HED

The results of PHF supplementation on performance parameters in broilers fed HED were evaluated on days 35 and 42 and are presented in Tables 4 and 5. There was no difference in the BW of birds among the groups before the initiation of the experiment, indicating the homogenous nature of the groups in terms of body weight. Numerically, the NC group had a lower BW than the other treated groups, albeit without any statistical significance on days 35 and 42. There was no difference in BW across the groups on day 35, except for PHF750, which showed a 15 gm BW improvement compared to NC. However, SCC, PHF400, PHF500, and PHF750 all produced a numerical and noticeable improvement in BW on day 42; the PHF750 supplemented group had a similar BW compared to the SCC 1,500 gm/ton group on day 42.

**Table 2.** Feed formula—grower (HED—NC group).

Feed Ingredients	Quantity (gm/kg)	Calculated nutrient value	
Maize	630.00	ME kcal/kg	3100.0
Soybean meal 50	300.00	Crude protein %	21.0
De-oiled rice bran	10.70	**Digestible amino acids %**	
Rice bran oil—Finisher^a^	25.00	Lysine	1.15
Di-calcium-phosphate	13.10	Methionine	0.45
Limestone powder	6.30	Methionine + Cysteine	0.85
DL-Methionine	2.70	Threonine	0.78
L-lysine HCl	3.00	Tryptophan	0.20
L-threonine	1.50	Arginine	1.20
Salt	2.00	Isoleucine	0.75
Sodium-bi-carbonate	2.00	Valine	0.85
Trace mineral premix^b^	0.50	Calcium %	0.75
Antibiotic Growth promoter	0.50	Available P %	0.45
Vitamin Premix^c^	0.50	Crude fiber %	2.81
Toxin Binder	1.00	Crude fat %	5.74
Mould inhibitor	0.50	Sodium %	0.22
Salinomycin 12%	0.50	Chloride %	0.19
Phytase 5000 EC	0.10	Potassium %	0.86
NSPase	0.10	Choline ppm	1250

SCC and PHF at defined doses were added by replacing the equal quantity of de-oiled rice bran in HED.

a ME value assigned to rice bran oil was 8800 kcal/kg.

The ^b^mineral and ^c^vitamin premix were prepared as per the composition [8].

Moreover, FCR in PHF500 and PHF750 was better than NC and SCC groups on days 35; SCC and PHF displayed a better FCR than NC at all doses; PHF750, and SCC 1500 showed a comparable FCR on days 42. Similarly, livability and EPI improved in SCC and PHF supplemented groups compared to NC, and the improvement observed in PHF750 was on par with SCC 1500. 

### Impact of PHF on protein and fat accretion in broilers fed HED

The results of PHF and SCC supplementation on the protein and fat accretion in broiler chickens are specified in Table 6. No statistically significant difference was noticed in protein accretion across the groups, yet numerical improvement was observed in the SCC and PHF supplemented groups. However, fat accumulation was significantly reduced in the SCC, PHF400, PHF500, and PHF750 groups compared to NC.

### Impact of PHF on serum L-carnitine levels in broilers fed HED

**Figure 1.**
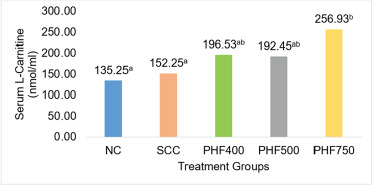
Impact of PHF on serum L-carnitine levels in broilers fed HED. Data are expressed as the mean of 3-5 birds from each dietary treatment. ^ab^Values with dissimilar superscripts differ significantly (*p* < 0.05).

The outcome of PHF and SCC supplementation on serum LC levels of broilers fed HED is demonstrated in Figure 1*.* PHF750 produced a statistically significant increase in serum LC levels compared to other treatment groups, whereas SCC, PHF400, and PHF500 showed a numerical increase compared to NC. 

## DISCUSSION

The fat is mobilized in low-density lipoproteins from hepatic to extrahepatic cells, where they get metabolized or accumulated, mediated by an essential lipotropic factor, choline [21]. Consequently, the excess energy from fat gets diverted more towards the accretion of muscle protein and body mass than carcass fat, resulting in better performance traits and lower lipid content in the carcass and visceral organs [22]. Typically, L-carnitine helped the beta-oxidation process by making it easier for long-chain fatty acids to move into the inner mitochondrial matrix. Although the metabolic role of L-carnitine has been widely evaluated in other species, its role in the production performance and meat quality of commercial broilers is less understood. So, this study was set up to look at how PHF and SCC affect growth, protein, and fat accretion in the carcass and serum LC content in Cobb 430 broilers fed a lot of HED.

**Table 3.** Feed formula—finisher (HED—NC group).

Feed Ingredients	Quantity (gm/kg)	Calculated nutrient value	
Maize	657.00	ME kcal/kg	3200.0
Soybean meal 50	270.00	Crude protein %	19.0
De-oiled rice bran	10.70	**Digestible amino acids %**	
Rice bran oil—Finisher^a^	32.00	Lysine	1.05
Di-calcium-phosphate	10.60	Methionine	0.40
Limestone powder	5.60	Methionine + Cysteine	0.80
DL-Methionine	2.50	Threonine	0.74
L-lysine HCl	2.40	Tryptophan	0.18
L-threonine	1.50	Arginine	1.10
Salt	2.00	Isoleucine	0.70
Sodium-bi-carbonate	2.00	Valine	0.80
Trace mineral premix^b^	0.50	Calcium %	0.70
Antibiotic Growth promoter	0.50	Available P %	0.42
Vitamin Premix^c^	0.50	Crude fiber %	3.00
Toxin Binder	1.00	Crude fat %	5.43
Mould inhibitor	0.50	Sodium %	0.22
Salinomycin 12%	0.50	Chloride %	1.90
Phytase 5000 EC	0.10	Potassium %	0.80
NSPase	0.10	Choline ppm	1,150

SCC and PHF at defined doses were added by replacing the equal quantity of de-oiled rice bran in HED.

a ME value assigned to rice bran oil was 8800 kcal/kg.

The ^b^mineral and ^c^vitamin premix were prepared as per the composition [8].

**Table 4.** Impact of PHF on zootechnical parameters in broilers fed HED.

Group	Body weight (gm)			FCR		Livability (%)	EPI
	1st day	35th day	42nd day	35th day	42nd day	42nd day	42nd day
NC	44.8	2292.1	3154.3	1.522	1.641	90.0	411.8
SCC	44.8	2293.8	3194.3	1.532	1.616	94.0	443.4
PHF400	44.8	2292.7	3177.1	1.531	1.637	95.0	439.1
PHF500	44.8	2298.2	3178.8	1.518	1.624	94.0	438.1
PHF750	44.8	2307.2	3195.9	1.493	1.606	95.0	450.1
SEM	0.02	7.01	6.78	0.005	0.005	0.844	4.17
*p*-value	0.931	0.907	0.864	0.522	0.490	0.516	0.196

SEM—Standard error of means.

In this study, the protein content in muscle and the protein accretion in the carcass was not significantly influenced by the dietary interventions. Yet, numerical improvement was observed in the SCC and PHF supplemented groups. However, the fat content in the NC group’s carcass was higher than other dietary interventions. A similar trend was noticed in the case of fat accretion in the carcass during 1–42 days of age, with the NC group showing significantly higher fat accumulation compared to the remaining dietary groups (p < 0.05). This result suggests that in the absence of supplemental choline or PHF, the experimental birds deposited more fat in the carcass, indicating poor metabolic efficiency where more energy is required to convert nutrients into fat rather than protein or body mass. The reduction in fat and fat accretion of the carcass observed in the SCC and PHF supplemented groups could be attributed to their lipotropic action; this is in accordance with literature findings reporting the reduction in abdominal and liver fat when broilers were fed a diet supplemented with an herbal source of choline [23]. Saeed et al. [24] reported that including herbal formulations and lipotropic agents in the feed reduces the adverse metabolic implications of a high-energy diet in poultry. Moreover, PHF at 400 gm/ton feed reduced abdominal fat and liver fat in broilers fed a low choline diet [8]. 

**Table 5.** Impact of PHF on CFI and average daily feed intake in broilers fed HED.

Group	CFI (gm)		Average Daily Feed Intake (gm)	
	Day 1–35	Day 1–42	Day 1–35	Day 1–42
NC	3489.4	5102.4	99.70	121.49
SCC	3514.2	5089.3	100.40	121.17
PHF400	3505.9	5128.3	100.17	122.10
PHF500	3538.6	5090.4	101.10	121.20
PHF750	3503.4	5058.1	100.10	120.43
SEM	13.71	15.21	0.376	0.36
*p*-value	0.798	0.804	0.797	0.804

SEM—Standard error of means.

**Table 6.** Impact of PHF on accretion of the protein and fat in broilers fed HED.

Group	Carcass N_2_		Carcass Fat		Protein Accretion		Fat Accretion	
	gm/100 gm	Total (gm)	gm/100 gm	Total (gm)	Total (gm)	gm/day	Total (gm)	gm/day
NC	3.72^a^	117.73^a^	0.88^b^	28.04^b^	730.53^a^	17.39^a^	26.37^b^	0.628^b^
SCC	3.69^a^	118.64^a^	0.64^a^	20.59^a^	736.25^a^	17.53^a^	18.93^a^	0.451^a^
PHF400	3.80^a^	120.65^a^	0.66^a^	21.04^a^	748.82^a^	17.83^a^	19.37^a^	0.461^a^
PHF500	3.69^a^	117.83^a^	0.67^a^	21.25^a^	731.32^a^	17.41^a^	19.58^a^	0.466^a^
PHF750	3.73^a^	120.12^a^	0.67^a^	21.66^a^	745.54^a^	17.75^a^	20.00^a^	0.476^a^
SEM	0.087	0.785	0.023	0.736	13.02	0.28	0.735	0.015
*p*-value	0.702	0.642	0.04	0.049	0.645	0.623	0.049	0.049

Data are expressed as the mean of 4-5 birds from each dietary treatment.

^ab^Values with dissimilar superscripts in a column differ significantly (*p* < 0.05).

SEM—Standard error of means.

Furthermore, the dietary groups supplemented with PHF400, PHF500, and PHF750 revealed higher serum LC levels than SCC and NC. The improvement in serum LC levels displayed by PHF400, PHF500, and PHF750 was 45.3%, 42.3%, and 90%, respectively, comparatively more significant than SCC (12.6%). Our results are in concordance with the reports wherein supplementation of L-carnitine significantly improved the production performance of chickens during the early stages of chicken growth and decreased the abdominal fat during the finisher period [25]. L-carnitine plays a critical role in energy generation as it shuttles the long-chain acyl-CoA to the inner mitochondrial membrane for beta-oxidation [26]. In addition, L-carnitine is reported to increase the activity of the enzyme hepatic carnitine palmitoyltransferase and decrease the concentrations of serum triacylglycerol and non-esterified fatty acid in chickens [27], as well as decrease lipid accretion in the carcass of young pigs [28]. 

In the current study, the birds grew quite well by day 42, and the average BW was more than the breed standard for male birds (3,044 gm). As a result, the feed intake (Table 5) was higher and more than the breed standard (5,073 gm in 42 days). Regarding BW, feed consumption was similar to that of other dietary treatment groups. Only subtle differences were observed between the groups concerning the ADFI calculated during the starter, grower, and finisher stages. In comparison with NC, the supplementation of SCC (1,500 gm/ton) and PHF (400, 500, and 750 gm/ton) improved the BW by about 40, 22.8, 24.5, and 41.6 gm, respectively, and consumed 25, 4, 17, and 35 gm less feed per unit BWG, respectively. Statistical insignificance notwithstanding, some interesting numerical differences were observed in this study. The PHF750 group was found to have the highest BW across all the treatment groups while consuming the lowest amount of feed-in 42 days. This result is in concordance with the findings of Farina et al. [29], Ryan et al. [30], and Emmert and Baker [31], who observed the dose-response of choline on BWG of birds fed a low choline diet. These findings affirmed that the positive response of birds’ BW and feed efficiency to choline supplementation and natural lipotropic agents could be effectively used as a good indicator of efficacy in broiler chickens fed HED.

Furthermore, livability varied between the treatment groups. Mortalities observed during the experimental period were mostly accidental, and post-mortem examinations did not reveal any specific pathognomonic lesions to identify the cause of the mortality. Nevertheless, SCC and PHF supplementation in HED produced better livability that was 4%–5% higher than NC. In addition, SCC and PHF supplemented groups also displayed a better EPI owing to higher BW, improved FCR, and better livability. However, a comparable or relatively better EPI was observed in PHF750 compared to SCC (450.1 *vs*. 443.4). This result signifies that PHF at 750 gm/ton feed effectively replaces 1,500 gm SCC in the broiler ration and exhibits a similar SCC performance as reflected by EPI. Calderano et al. [10], Khose et al. [16], and Khosravinia et al. [32] showed an improvement in survivability, feed efficiency, and body weight when a natural source of choline was used to substitute synthetic choline in bird diets, and this agrees with our study findings.

The liver is involved in many homeostatic and metabolic functions and is considered a biochemical factory responsible for the detoxification, metabolism, and excretion processes in birds and mammals [33]. Nutritional modifications affect the liver and influence the nutrient metabolism in avian species. Thus, we have prepared the herbal formulation comprising A. nilotica bark and C. longa plant parts, demonstrated choline-like functions in broilers fed a choline-deficient diet [8]. These individual plants, rich sources of polyphenols and curcuminoids, respectively, were proven to have hepatoprotective activity in rats. In another study, A. nilotica was proven to possess a hepatoprotective effect, ameliorating acetaminophen-induced hepatotoxicity in rats [34]. The addition of C. longa extract increases the liver betaine content. It improves liver lipid metabolism by affecting the transmethylation pathway and the osmotic regulation of hepatocellular hydration, thereby protecting the liver from different stressors [35]. Chandrasekaran et al. [36] confirmed the lipotropic and anti-lipogenic activity of PHF and SCC in the rat model of methionine-choline deficiency (MCD) diet-induced hepatosteatosis and the oleic acid-induced lipogenesis assay in HepG2 cell lines, respectively. The PHF consisting of A. nilotica and C. longa is reported to positively modulate specific genes involved in growth promotion and improve the performance parameters in broilers fed a choline-deficient diet [37]. These observations suggest that the hepatoprotective, lipotropic, and anti-lipogenic activity of PHF contributes to improved growth parameters. At the same time, the decrease in carcass fat accretion in the current study could be attributed to elevated serum L-carnitine levels. The results from this study ascertain that the PHF under investigation (Kolin Plus™) positively influences the performance of broilers similar to SCC by improving the fat metabolism and mobilization by enhancing the serum LC levels and restoring normal fat accretion and better performance traits. Therefore, Kolin Plus™ can be explored as a substitute for SCC in poultry diets.

## Conclusion

The supplementation of PHF in HED significantly increased serum L-carnitine levels. It decreased fat accretion, indicating an improved fat metabolism and mobilization with a subsequent increment in the zootechnical parameters of broilers. Kolin Plus™ supplementation improves the performance traits of broilers similar to SCC by improving fat metabolism and mobilization by enhancing serum LC levels and restoring normal fat accretion. Moreover, PHF at 750 gm/ton feed could replace 1,500 gm/ton of choline chloride (SCC, 60%) in high-energy broiler feeds. Though there is a lack of an exact correlation between carcass fat content and serum L-carnitine concentration, closer scrutiny reveals that perhaps with lower serum L-carnitine levels, fat accretion was higher. However, further investigations are needed to elucidate the molecular mechanism of Kolin Plus™ and its role in fat metabolism and mobilization in chickens.

## List of abbreviations

PHF, Polyherbal formulation; LC, L-Carnitine; HED, High energy diet; NC, Negative control; SCC, Synthetic choline chloride; FCR, Feed conversion ratio; PVC, Polyvinyl chloride; BW, Body weight; EPI, European production index; CFI, Cumulative feed intake; ADFI, Average daily feed intake; BWG, Body weight gain; SEM, standard error of means.
